# Ethical Issues and Recommendations in Psychedelic Research and Practice: A Scoping Review

**DOI:** 10.1007/s11673-025-10454-3

**Published:** 2025-08-07

**Authors:** N. Brittain, N. Higgins, M. Barber, W. Choi, A. Carter, J. Gardner

**Affiliations:** 1 School of Psychological Sciences, Monash University, 770 Blackburn Rd, Clayton Melbourne, VIC 3800 Australia; 2 Brown University, Providence, RI 02912 USA; 3 Monash Bioethics Centre and the School of Philosophical, Historical and International Studies, School of Psychological Sciences, Monash University, 770 Blackburn Rd, Clayton, Melbourne, VIC 3800 Australia

**Keywords:** Psychedelic-assisted therapy, Psychedelic research, Ethics, Justice, Cultural appropriation, Equity

## Abstract

The rapid growth in psychedelic research raises novel ethical challenges for both research and psychedelic-assisted therapy. Despite these challenges, there is no consensus among researchers, clinicians, patients, and regulators on how these ethical issues may be avoided or managed. This study aimed to identify key ethical issues in psychedelic research and practice in the literature. A scoping review was performed, identifying fifty-one relevant articles. Content analysis revealed five main ethical concerns in the literature: 1) standards of practice, 2) equity, 3) integrity, 4) cultural appropriation, and 5) epistemic justice. A consultation workshop highlighted several key concerns that were neglected in the literature: 1) post-trial care, 2) lack of consensus on models of care and psychedelic practitioner competencies, and 3) how current research and its associated ethical issues will be translated into practice. Based on these findings the following future directions are recommended: designing clear competency frameworks, increased consultation with Indigenous and priority populations (e.g. ethnic and cultural minorities, under-served groups), guidelines for Indigenous recognition, management of post-trial care in clinical trials, and adequate governance of psychedelic prescribing practices.

**Supplementary Information:**

The online version contains supplementary material available at 10.1007/s11673-025-10454-3.

## Introduction

“Psychedelic” refers to a class of psychoactive substances that produce altered states of consciousness (Vollenweider and Preller 2020), including both serotonin agonists (e.g., psilocybin) and drugs with diverse neurochemical effects (e.g., ketamine, MDMA). There has been a resurgence in psychedelic research to understand and treat mental illness in the last decade (Hall 2022). Psychedelic-assisted therapy (PAT) is an intensive therapeutic intervention which involves a programme of preparation, psychedelic-facilitated dosing sessions, and integration (Schenberg 2018). Positive outcomes from trials of PAT have been reported for the treatment of depression and post-traumatic stress disorder (PTSD) (Carhart-Harris et al. 2021; Mitchell et al. 2021). There has been a surge in public and media interest in psychedelic research and investment by philanthropic and commercial organizations following these promising early-stage results. The psychedelic drug market is predicted to reach around USD $11 billion by 2027 (FinancialNewsMedia.com 2021), and the commercialization of PAT has expanded access to psychedelic drugs (Smith and Appelbaum 2021). However, many common psychedelic substances utilized in research are illegal to possess in many jurisdictions (Therapeutic Goods Administration 2024), adding to the complexity of ethical considerations in clinical and research settings.

Although consumer access to PAT is rapidly increasing worldwide (Smith and Appelbaum 2021), research into what constitutes ethical and safe incorporation of psychedelics into therapy is lacking (McNamee et al. 2023). The use of psychedelics as medicines in clinical practice raises novel ethical concerns that may be difficult to capture within traditional informed consent processes. Psychedelic sessions induce an altered state of consciousness that can leave participants highly vulnerable and suggestible (Vollenweider and Preller 2020). The nature of this altered state is difficult to communicate to participants, particularly those without previous psychedelic experience (Smith and Appelbaum 2022; Smith and Sisti 2021). The common practice of providing “nurturing touch” to participants while under the influence of a psychedelic raises concerns about boundary-setting practices in vulnerable participants (MAPS 2015, 10; Smith and Appelbaum 2021). Another ethical issue raised by PAT is the appropriation of Indigenous knowledge and medicines, which separates them from their sacred cultural and spiritual contexts (Celidwen et al. 2023). Some have argued that current PAT research does not adequately recognize, respect, or compensate Indigenous communities for the appropriation of their knowledge in Western psychedelic research and practice (Bouso and Sánchez-Avilés 2020; Celidwen et al. 2023). Further, Indigenous people are often excluded from PAT clinical research (Celidwen et al. 2023; Michaels et al. 2018).

The push to commercialize psychedelics and increase access to PAT raises concerns about training standards, the prioritization of participants’ wellbeing over financial incentives (Plesa and Petranker 2022), and regulation. Publicly available PAT manuals and protocols provide some guidance for practitioners (e.g., Mithoefer 2017; Phelps 2017) but reflect the views and practices of key figures in early current-wave psychedelic research. There is currently no consensus on the minimum training requirements for PAT providers or how PAT should be provided (Kious et al. 2023). Short, psychedelic-specific training programs for therapists are being developed in response to the potential market for PAT and to meet increasing demand in the community for accredited PAT (Plesa and Petranker 2022). There is concern amongst some commentators, particularly those from underserved communities, that these programs may overlook issues such as the lack of diversity represented in trials of PAT (e.g., race, education, psychiatric, and medical comorbidities) and uncertainty regarding the minimum competencies needed to practice PAT safely and ethically (Celidwen et al. 2023; Plesa and Petranker 2022; Williams and Van Meter 2023). The emergence of for-profit ketamine clinics, reportedly operating with insufficient screening and monitoring of patients (Smith and Appelbaum 2021), and the surge in medicinal cannabis clinics despite limited evidence of efficacy beyond a small handful of indications, provide strong warnings of how PAT may evolve if not appropriately regulated (Dobson et al. 2024); Smith and Appelbaum 2021).

Recognizing the need for an overarching framework for ethical guidance in psychedelic research, Spriggs et al. (2023) recently published an Access Reciprocity and Conduct (ARC) framework, to be further developed in consultation with stakeholders. However, in a field that has significant vested interests, including those of pharmaceutical companies, commercial interests, and advocacy groups (Plesa and Petranker 2022), it is uncertain whether Indigenous and other diverse views are being adequately represented in psychedelic research and practice. A review of the existing discourse on ethical issues in psychedelic medicine to assess the strengths and gaps in the literature is urgently needed in order to support overarching frameworks and ensure the responsible innovation of psychedelic products. To address this need, we conducted a scoping review of the current literature, identifying key ethical issues and guidelines in psychedelic research and practice, and highlighting those requiring further attention.

## Methods

A scoping review was performed to identify key ethical issues and recommendations for ethical psychedelic research and practice. The study followed the six steps outlined by Levac et al. (2010) and is reported according to the Preferred Reporting Items for Systematic Review and meta-analysis-Scoping Review extension (PRISMA-ScR) protocol (Tricco et al. 2018; Supplementary Material A).

### Identification of Relevant Studies

A systematic search was conducted between 14 and 24 April, 2023, in PsycINFO (Ovid), ProQuest, Medline (Ovid), and Scopus (Elsevier) (see Table 1 and see Supplementary Material B for full electronic search history).

**Table 1:** Search Terms for PsychINFO *(these were adjusted for use in Proquest, Medline and Scopus)*

Concept - Ethics/Guidelines
• Ethics (Includes bioethics, experimental ethics, professional ethics, business ethics, consumer ethics, plagiarism) • OR Treatment Guidelines • OR ethic* • OR guideline* • OR framework* • OR *transparency* • OR competence*
Concept - Psychedelics
• Psychedelic Drugs • OR ketamine/OR lysergic acid diethylamide/OR mescaline/OR methylenedioxymethamphetamine/OR psilocybin/OR mescaline/OR bufotenine/ • OR *ayahuasca* OR *ibogaine* OR *N,N-Dimethyltryptamine* • OR Psychedelic Assisted Therapy • OR psychedeli* • OR hallucinogen*

Italicized are keywords, non-italicized are MeSH terms or subject headings.

### Selection of Studies for Inclusion

**Figure 1:**
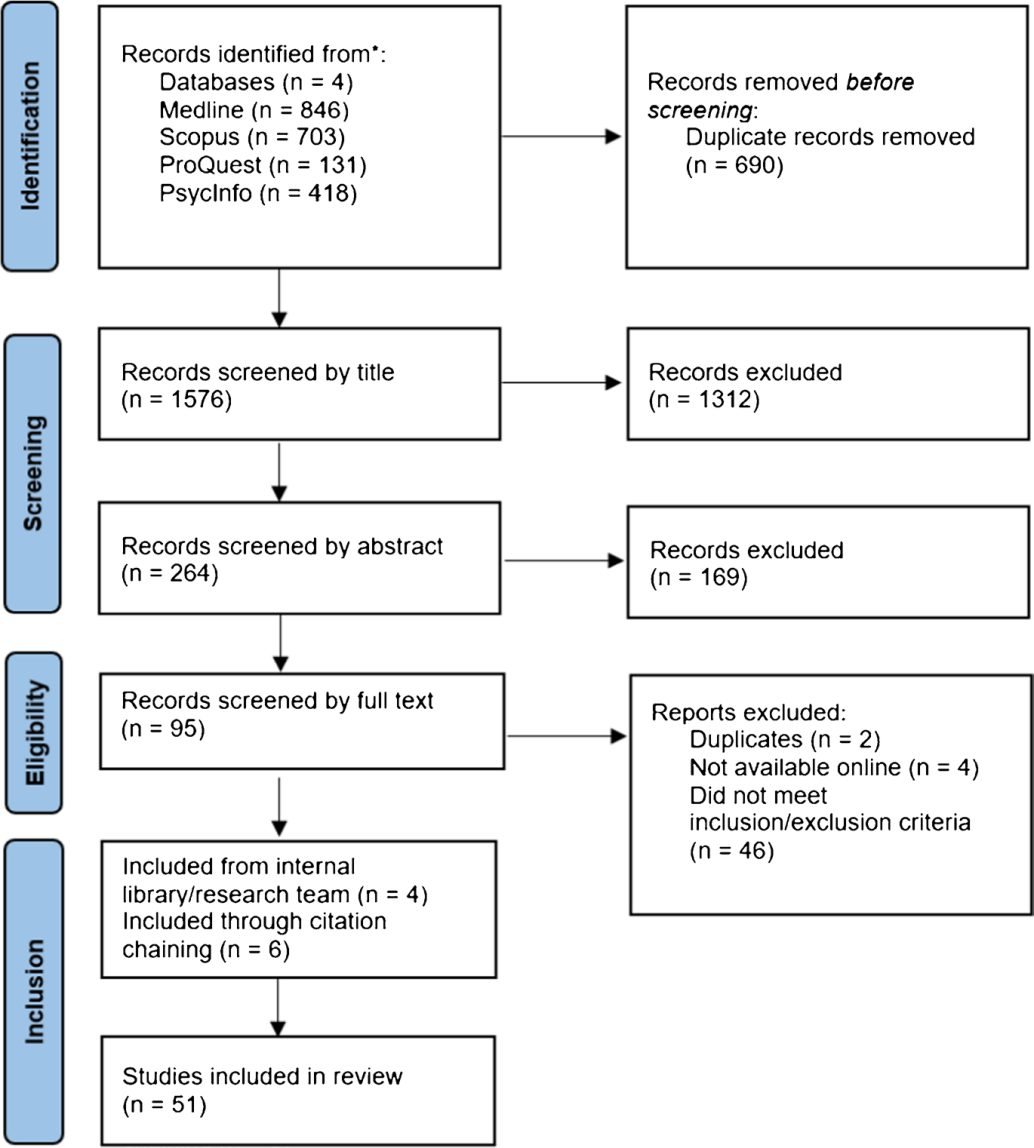
Identification of Studies via Databases

Studies were included in the analysis if they 1) were in English, 2) dedicated a substantive portion of the article to discussion of the ethical issues in psychedelic therapy or research, and/or 3) included recommendations for the ethical practice of psychedelic therapy or research. Studies were excluded if they focused on 1) the clinical safety and efficacy of PAT or 2) the non-psychotherapeutic use of psychedelics (e.g., recreational use, ketamine intranasal spray, or anaesthetic use). NB screened the articles for inclusion by title, abstract, and full text (see Figure 1). A random sample of 10 per cent of papers were screened by NH at the title and abstract stage, and a further 20 per cent were screened by full text. Agreement between reviewers at this stage was 90 per cent. Disparities were discussed in team meetings to achieve consensus. Citation chaining and snowball sampling were used to identify additional articles. Data collection finished on July 28, 2023.

### Data Analysis

A content analysis (Hsieh and Shannon 2005) of the final dataset was conducted in NVivo (QSR International Pty Ltd 2020). Analysis began with open coding of the data, focusing on passages that identified and discussed ethical issues in PAT research and practice (Gale et al. 2013). Codes were then grouped together based on thematic similarity to form a working analytical framework that was adjusted as new concepts were identified (Gale et al. 2013). Discrepancies in the interpretation of the data were discussed amongst the research team until consensus was reached.

### Charting the Data

**Figure 2:**
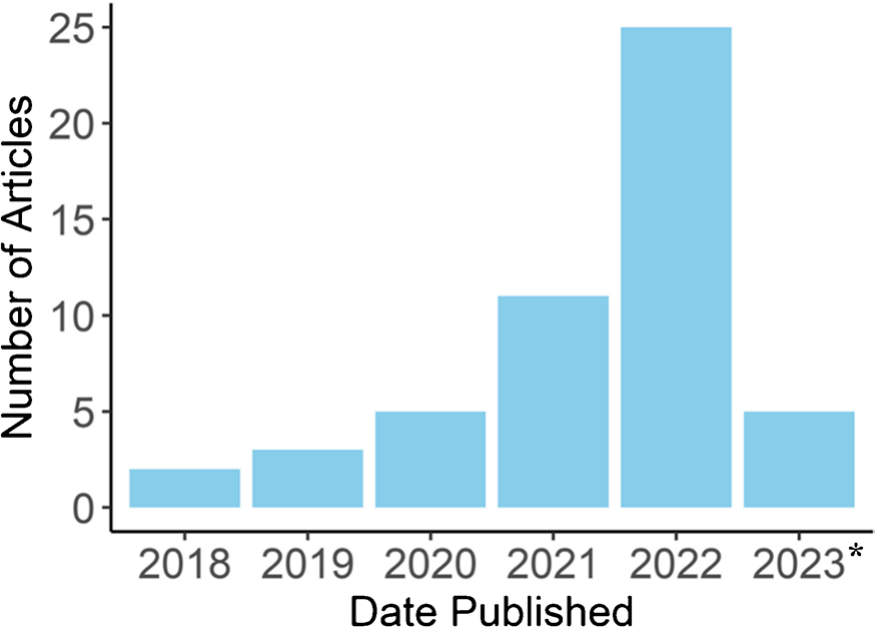
Articles by Publishing Date. Note: *2023 spans January to end of June

**Figure 3:**
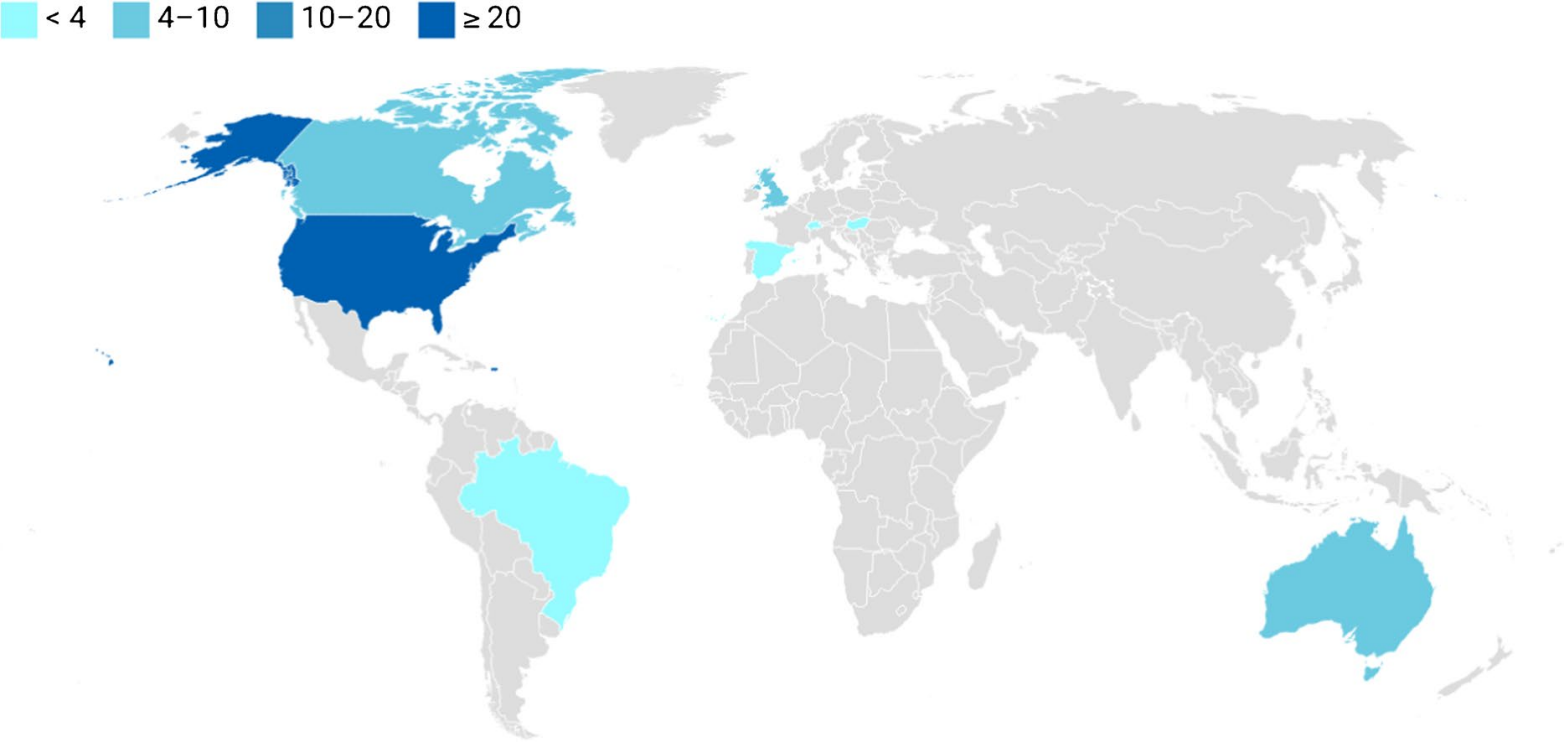
Geographic Distribution of Articles Published by Institution of First Author

Key bibliographic information was extracted from the data (Arksey and O'Malley 2005), including article type, publishing date, organizational affiliation, and location of the first author. We also reported engagement with lived experience expertise (e.g. PAT therapists and participants of PAT trials), Indigenous peoples or other stakeholders, and other relevant author declarations (e.g. commercial interests). See Figures 2 and 3. Ethical themes identified through content analysis were charted in Table 2.

**Table 2:** Ethical Issues Identified in the Literature

Ethical Issue	Description	Number of Articles ^a^	Articles Cited ^b^
1. Standards of Practice ***Non-Maleficence and Beneficence***	Researchers and therapists’ obligation to abstain from causing harm to participants and to promote participants welfare.	42 33	1, 2, 4, 6, 7, 8, 9, 10, 11, 12, 13, 16, 20, 22, 24, 25, 27, 29, 30, 32, 33, 34, 36, 38, 40, 42, 43, 44, 45, 47, 49, 50, 51
***Competence***	The skills and training needed to practise*.*	21	2, 4, 8, 9, 10, 11, 14, 16, 24,26, 30, 32, 36, 37, 40, 42, 43, 45, 46, 47, 48
***Informed Consent***	The practice of informing participants prior to making decisions about their healthcare.	16	2, 5, 6, 9, 11, 20, 23, 29, 30, 32, 34, 40, 43, 44, 45, 47
***Therapist Attributes***	The qualities practitioners possess.	24	1, 2, 4, 7, 8, 9, 11, 14, 21, 22, 24, 26, 27, 29, 30, 31, 32, 36, 37, 40, 43, 44, 45, 47
2. Equity	The fair and just treatment of all patients and research participants.	35	1, 3, 4, 5, 6, 10, 12, 13, 14, 15, 16, 17, 19, 25, 26, 27, 28, 29, 30, 32, 33, 34, 36, 37, 38, 39, 40, 41, 42, 43, 45, 46, 48, 49, 50
3. Integrity	Ethical practice in research.	31	1, 2, 4, 5, 6, 9, 11, 13, 14, 17, 18, 19, 21, 22, 25, 29, 30, 32, 34, 35, 36, 37, 40, 41, 43, 44, 45, 46, 48, 49, 51
4. Cultural Appropriation and Safety	The extraction of Indigenous plants and knowledge without consent.	23	7, 10, 13, 14, 15, 16, 17, 19, 24, 25, 26, 28, 29, 32, 36, 39, 40, 41, 43, 45, 46, 48, 49
5. Epistemic Justice	A form of harm that can include the silencing, distortion, or mistrust of certain ways of knowing.	15	7, 15, 16, 19, 23, 24, 25, 30, 31, 37, 39, 40, 41, 49, 50

^a^ Refers to the number of articles that mentioned each ethical issue

^b^ Numbers provided in these columns refer to the article reference number included in Supplementary Material C

### Summarizing Results

The findings of the content analysis and descriptive numerical summaries are presented in a narrative form (Arksey and O’Malley 2005), and the viewpoints represented in the literature are characterized by article bibliographical details. Ethical issues that were not adequately addressed in the literature were identified based on their frequency relative to the discussion of other issues, and through feedback on the results from a consultation workshop.

### Consultation

An interdisciplinary consultation workshop (following Levac et al. 2010) was conducted to further explore our findings from key stakeholder perspectives (Lincoln and Guba 1985). Eight individuals from psychedelic research, healthcare, and the commercial sector participated in a ninety-minute online workshop that examined the following: 1) Are these ethical issues consistent with your own experience as a psychedelic researcher or practitioner? 2) Are there any issues that you found particularly pertinent, and if so why? 3) What ethical issues have not been adequately represented in these findings? 4) Do you have any recommendations for addressing these ethical issues that were not identified in the literature? Responses to these questions were used to refine our analysis of the results.

## Results

### Sample Characteristics

The search identified fifty-one articles discussing ethical issues or practice guidelines in psychedelic research and therapy (see Supplementary Material C). These have been assigned a numerical citation, which have been used throughout the results to refer to key examples of the ethical issues discussed.

All included articles were published in the last five years (see Figure 2). Most articles were led by authors from the United States (55.8 %; Figure 3), Canada (11.5 %), UK (9.6 %), and Australia (7.7 %). Additional articles were published by authors in Brazil, Switzerland, Spain (3.8%), and Hungary (1.9%).

Only nine articles employed empirical methods to understand the ethics of PAT, including five that engaged with stakeholders. Of these, one was led by Indigenous authors (13), one consulted Indigenous stakeholders (45), and two consulted individuals with lived experience—previous trial participants and PAT therapists (9, 14). Two articles also consulted research, industry, and community stakeholders (14, 45), and one article consulted experts in psychology, ethics, religious studies, palliative care, anthropology, and legal studies (40).

Thirteen articles included declarations that one or more of the authors were in paid advisory relationships or receiving other financial support from psychedelics industry/organisations (e.g., COMPASS, Silo Pharma). One author declared that they were a member of an ayahuasca religion (4).

### Content Analysis

Five ethical themes were identified through the content analysis: 1) standards of practice (which included sub-themes of beneficence/non-maleficence, competence, informed consent, and therapist attributes), 2) equity, 3) integrity, 4) cultural appropriation, and 5) epistemic justice (see Table 2). There was diversity in the frequency and depth of each article’s discussion of the issues captured within the theme, and the recommendations made to deal with these ethical challenges.

#### Standards of Practice

##### Non-Maleficence and Beneficence

The principle of non-maleficence in this context refers to researchers and therapists’ obligation to abstain from causing harm to participants. Non-maleficence was identified in thirty-three articles, and was primarily discussed in terms of the physiological, emotional, and psychological side effects culminating during altered states (e.g., 11, 40, 50). Several articles suggested that potential adverse effects were not fully understood and potentially underreported and underestimated, such as increased heart rate, breathing rate, and psychological distress (e.g., 29, 40). Articles also highlighted the increased vulnerability participants experience during altered states. Vulnerability was described as stemming from the imbalanced power dynamic between practitioner and client, an enhanced sense of intimacy (e.g., 9), participants’ heightened emotional sensitivity and increased suggestibility whilst under the influence of psychedelics (e.g., 1, 47), and the fact that the participant cannot leave the premises during the acute psychedelic experience (e.g., 10).

Several articles suggested that PAT had a favourable risk-benefit ratio (e.g., 2, 44). However, authors generally highlighted the need for more research with priority populations such as Black, Indigenous and people of colour (BIPOC), LGBTQIA+ people, and people with disabilities in order to assess the safety of PAT in these populations (e.g., 6, 32, 38). One article suggested that risk-benefit analyses ignore the challenges individuals from priority populations may face in consenting to PAT, due to factors such as cognitive disabilities, marginalization, or vulnerability to coercion (34).

Beneficence in this context refers to researchers and therapists’ obligation to promote participants’ welfare. Beneficence was only discussed in a few articles, with a focus on the moral obligation to provide treatment to those in need (e.g., 49, 33). One article mentioned post-trial access (6), expressing concern that the legal status of psychedelic drugs constituted a barrier to participants being able to benefit from PAT post-trial.

Suggestions for upholding the principles of non-maleficence and beneficence in PAT research and practice included advising participants of safety guidelines prior to the session (e.g., 40), providing post-session integration care, and providing a safe and supportive “setting” (e.g., 6) (“setting” refers to the physical and social environment of sessions [Schenberg 2018]). One article suggested a public-private partnership, where stakeholders collaborate to create and promote best practice standards for harm reduction, risk mitigation, safety monitoring, and benefit maximization, as well as provide recourse for individuals harmed during PAT (4). Suggested stakeholders were government and non-governmental departments and organisations, representatives of Indigenous communities that traditionally use psychedelic substances, academic researchers, and sponsors of drug development (4).

##### Competence

Competence was discussed in twenty-one articles and highlighted the importance of practicing within one’s area of expertise or training (e.g., 2, 37, 40). Several articles expressed concern that there was no legal credentialing system for PAT practitioners (9, 45). Many articles indicated that cultural safety and/or humility are important competencies in PAT (e.g., 10, 14, 36, 45), including an understanding of racial trauma (42, 16). Strategies for supporting competent and culturally safe practice included having therapist teams who matched the ethnicity of the client (10), having two therapists (43), and creating a culturally safe setting (48, 26). One article also suggested incorporating shamanic practices, which may include healing songs, prayer, and the creation of a personal altar for the participant (36).

##### Informed Consent

Informed consent was discussed in sixteen articles and revolved around three main topics: 1) gaining informed consent prior to psychedelic sessions, 2) informing participants of the risks and ineffability of the psychedelic experience, and 3) safely engaging in therapeutic touch. Authors questioned whether truly informed consent for PAT could be gained prior to psychedelic sessions (e.g., 9, 40, 45). Reasons driving this doubt were that participants have unrealistic expectations due to media hype (e.g., 20, 43), participants may experience pressure to participate from enthusiastic researchers (22), and the ineffability of the psychedelic experience could not be adequately conveyed to participants prior to the session (e.g., 20, 32, 44). Authors additionally questioned whether informed consent could be gained from individuals with previous psychedelic experience who may underestimate harms (6), and participants with cognitive disabilities, substance addiction, or communication impairments (e.g., 6, 32, 34). The issues with surrogate consent were discussed in circumstances where the participant does not have capacity to consent, such as those with disorders of consciousness (34). One article mentioned the potential for “therapeutic misconception” in PAT research, where participants may not understand that the primary aim of a trial is to advance scientific knowledge and not to directly benefit the participant (6).

Articles suggested several ways of conveying the ineffability and possible harms of the psychedelic experience. These were audio-visual aids (6), sharing testimonials from previous persons who have undergone a psychedelic experience (6, 32), using accessible and agential language (6, 30), and being clear about the evidence base for PAT (43). Authors also suggested making it clear prior to the session that participants could not leave part way through a psychedelic session for safety reasons (6, 43). One article suggested that fully informing participants of an ineffable experience was unrealistic and that simply informing them that it was “epistemically inaccessible” was adequate (20). Several articles expressed the need for standardized informed consent protocols (e.g., 23, 43).

Therapeutic touch, which is often employed during PAT, involves a practitioner providing comforting touch such as hand holding during the psychedelic session and raises additional challenges for informed consent (Smith and Appelbaum 2021). Concern was expressed over whether informed consent for therapeutic touch could be gained during altered states (e.g., 9, 40, 45). One author described how participants may consent to one option then change their mind during therapy and how assessing whether a client had decision-making capacity during an altered state may be counter-therapeutic (44). Suggestions included a two-stage process for therapeutic touch where consent (or lack thereof) was determined before the session and then re-ascertained during the session (9), only providing therapeutic touch when it is requested (44) and thoroughly documenting consent (11).

##### Therapist Attributes

Twenty-four articles discussed the values, morals, and competencies that PAT practitioners should possess. The importance of respect was discussed in terms of not discriminating against clients based on gender, religious beliefs, or other personal factors (e.g. 34, 36, 40). One article suggested that practitioners should cultivate spiritual intelligence (31) in order to increase empathy with participants who may have spiritual experiences during sessions. Another article suggested that therapists should not impose any spiritual beliefs on clients (21). Many articles highlighted the need to be empathetic, compassionate, and supportive as well as capable of building a therapeutic alliance and a trusting relationship with clients (e.g., 30, 31, 36).

The importance of self-awareness when practicing with vulnerable participants during altered states was highlighted and included being aware of increased transference and countertransference in the session (e.g., 8, 36, 40). Trust was suggested to be particularly important for BIPOC and other priority populations, including people who identify as LGBTQIA+ or with disabilities (e.g., 16, 27). The importance of supervision and self-care was commonly discussed (e.g., 2, 8, 9, 40). Self-care included practices such as being aware of one's intentions and needs entering a session and undergoing therapy to minimize the possibility of transference and boundary transgressions. Articles emphasized the importance of supervision in improving professional judgement and accountability.

Many articles discussed therapist use of psychedelics. Authors suggested that therapists should have experience using psychedelics because it enhanced client trust (9) and increased therapist understanding of the clients’ experience (31, 40). One article (36) suggested that if psychedelics were not available legally, therapists should try other methods such as holotropic breathwork. In contrast, another article discussed the possibility that psychedelic use may not be a necessary aspect for training practitioners and that it may affect clinical judgement and objectivity (22).

Boundary transgressions were discussed in fifteen articles. Unclear boundaries between the therapist and client during the psychedelic session were frequently discussed as a possible source of harm that could lead to exploitation of clients or the formation of dual relationships (e.g., 40, 45, 51). While boundary transgressions were usually seen as occurring due to increased vulnerability and intimacy between the client and practitioner (e.g., 1, 9, 11), one article suggested that in situations where participants presented a risk of harm to themselves or others, physical interventions may be necessary (40). Suggestions for maintaining professional boundaries included being self-aware of the risk of transgressions occurring, setting clear expectations prior to the session (8, 9), documenting consent, reporting transgressions, and upholding accountability (4, 36). It was suggested that the risk of dual relationships developing should be mediated by paying attention to confidentiality, trust, communication, and boundary setting (40). One article suggested that “energetic” boundary setting (9), which involved being conscious of nonphysical and nonverbal communication, was something practitioners should be aware of.

#### Equity

Equity in this context refers to the fair and just treatment of all patients and research participants. Equity was discussed in thirty-five articles and was described in terms of 1) inclusivity in research (e.g., 14, 26, 48) and 2) equal access to PAT (e.g., 4, 10, 45).

Inclusivity in research was widely discussed (n = 21). Most articles described a need for greater inclusion of BIPOC (e.g., 14, 26, 28), with several articles also discussing the inclusion of LGBTQIA+ and disabled persons (e.g., 10, 16, 32). Authors also noted the importance of excluding certain individuals for safety reasons. Excluding high-risk individuals, such as those with a personal or family history of psychosis, was understood as a protective measure against possible adverse effects of PAT (32, 50). On the other hand, excluding these groups was seen as a barrier to access which would prevent them from benefiting from PAT (e.g., 1, 30, 50). In particular, excluding disabled individuals was seen as a potentially paternalistic view (32)

Access to PAT was widely discussed (n = 18). Articles described an “inverse-care law” in psychedelic research and practice, where those who need treatment the most may be least likely to be able to access that treatment (48). Financial burden on participants was a commonly discussed barrier to access (e.g., 30, 48, 50). Articles suggested that due to the high cost of treatment, as well as practical considerations such as transit costs and time taken off work, PAT may be unaffordable for many (e.g., 17, 38, 48). One article expressed concern that negative connotations around psychedelic drug use may create a barrier to access (5). For BIPOC specifically, barriers included social stigma and stereotypes around illicit drug use (16) and mistrust in the medical system due to previous exploitation and harm (45).

Lack of access and diversity in research is closely linked to concerns around fair distribution of benefits. Many articles expressed the need for research to directly benefit traditional-knowledge holders as well as ethnic minorities (e.g., 16, 28, 45). However, what “benefit” required was rarely discussed, with one notable exception (13). Celidwen et al (2023) suggested sharing benefits derived from the use of Indigenous medicines and practices with source communities as they deem appropriate.

Authors expressed concern that a lack of access may motivate individuals toward underground use (37). Suggestions for improving access included reducing the number and length of sessions, scheduling sessions outside of business hours, conducting assessment via telehealth, and compensating participants for their time and transit costs (30). One article suggested that the use of non-hallucinogenic psychedelics—i.e., altered psychedelic compounds with reduced hallucinogenic potential (Dunlap et al. 2020)—may increase accessibility because they can be administered at home over telehealth (38). Policy suggestions included the use of programmes such as coordinated registry networks and prescription drug monitoring systems to promote expanded access and to determine need (4). Authors also discussed destigmatizing PAT for all participants, but particularly for BIPOC who may view PAT as unsafe or stigmatizing (16, 42). Consideration of cultural variation in symptom presentation and targeted advertising and referrals from outpatient providers were suggested ways to increase BIPOC recruitment in research (16).

Many suggestions for improving access and inclusion highlighted the need for collaboration and increased diversity in research teams (e.g., 27, 39, 40). Articles discussed the need for collaboration with BIPOC and disabled communities (e.g., 26, 30, 36). Increased diversity in research teams was suggested to better understand the barriers to minority access, improve practitioner-client pairings, and reduce harm during sessions (26).

#### Integrity

Integrity was discussed in thirty-one articles and revolved around two main topics: 1) conflicts of interest and 2) psychedelic exceptionalism and hype. Many authors expressed concern about financial conflicts of interest in psychedelic research (e.g., 17, 37, 40). Articles suggested that vested interests would not prioritize participant wellbeing, safety, and equitable access, would “crowd out” marginalized voices (36), cause distress, and undermine Indigenous participation (17). Vested interests could also overlook socio-political factors that lead to mental illness (37) and engender questionable research practices (35, 37). One author questioned whether it is the job of researchers to call out bad practice and what they should do if they find results that don’t align with desires of financial stakeholders (35). They highlighted the prevalence of vested interests, describing the common occurrence of receiving anonymous offers for money and illegally acquired or synthetic psychedelics (35) to “speed research along.”

A second and related concern was “psychedelic exceptionalism” (21), which was described as contributing to “blind devotion” to the research (11). Psychedelic exceptionalism is the belief that psychedelic drugs are uniquely transformative or beneficial, and as such should be exempt from the ethical, clinical, and legal requirements that other drugs are subject to (Johnson 2021). Authors expressed concern that exceptionalism was leading to questionable research practices, abuses of power, researchers not being transparent about the risks of psychedelics, and findings being overstated and embellished in the media (e.g., 11, 18, 37). One article discussed whether researchers having previous experience using psychedelics may impact objectivity and good science practices. It suggested that having researchers in the team who haven’t had personal experience with psychedelics may alleviate this risk (22).

Many suggestions for integrity in research recommended adherence to the principles of open science (UNESCO 2022). These included open data, pre-registration, peer-review, study replication, transparency, and providing a clear description of the generalizability of the study in research papers (e.g., 18, 21, 35). Authors also discussed the need to mediate public expectations by using transparent and objective public education and press releases (4, 37) and stated that psychedelic advocacy organizations should maintain high evidence-based standards and eschew media hype (32). Many articles suggested the need for ethical oversight, policy safeguards, supervision, and legal accountability for practitioners (e.g., 2, 4, 43).

#### Cultural Appropriation and Safety

Cultural appropriation, which involves the extraction of Indigenous plants (25) and knowledge (19), was discussed in twenty-three articles. Concern was expressed about how the commercialization and appropriation of psychedelics would harm Indigenous people (e.g., 13, 15, 24). Authors also discussed the epistemic harms linked to appropriation (e.g., 24, 25); this theme will be expanded on in the next section.

Authors expressed concern over the patenting of psychedelic drugs and intellectual property regimes that extract Indigenous knowledge (e.g., 13, 17, 41). Appropriated knowledge has informed trial design, and this has often been done without any meaningful consultation, consent, or permission from Indigenous peoples (17, 19). Further, Indigenous people have not received adequate compensation or benefit from the use of their knowledge (10, 29). Appropriation may cause intergenerational distress that perpetuates mental health issues (17) and causes spiritual practices to lose their meaning (15). One article described concern that shamans would be forced to align with new safety regulations (24). Other authors expressed concern that the deforestation of rainforests was occurring due to the extraction of natural plant medicines (13). This practice may leave Indigenous users without access to traditional medicines (7, 25).

Many articles identified the need to “recognize” (7, 19) and “acknowledge” (13, 26, 28) the Indigenous origin of psychedelic practice and for “reciprocity” (15, 36) and “respect” (7, 15), without providing specific recommendations for how this should be done. One article (13) did provide ethical principles and guidelines for Western psychedelic practice, namely “Reverence, Respect, Responsibility, Relevance, Regulation, Reparation, Restoration, and Reconciliation.” These principles were developed through Indigenous-led collaboration with stakeholders. Suggestions included acknowledgement of Indigenous traditions; protecting Indigenous intellectual property; free, prior, and informed consent in research; increased accountability for harmful practices; restoring Indigenous authority; safeguarding self-determination; active inclusion of Indigenous knowledge holders and practitioners; formal efforts to include Indigenous-led intellectual foundations in Western practice; benefit sharing; and reparation (13). Other suggestions in the literature included incorporating Indigenous methods (7), investing in Indigenous-led research (7), increasing collaboration in research and practice (48), sustainable harvesting of psychedelic plants (25), and practicing restorative justice (43).

#### Epistemic Justice

Epistemic injustice is a form of harm that can include the silencing, distortion, or mistrust of certain ways of knowing (Fricker 2007). This theme was identified in fifteen articles. Authors expressed concern that psychedelics were becoming primarily framed by Western biomedical approaches to epistemology. Most discussion around epistemology was concerned with Western epistemologies delegitimizing Indigenous knowledge and practices. Some have argued that Western epistemologies tend to be reductionist and objective, whereas many Indigenous epistemologies tend to be subjective and holistic (Antoine et al. 2018; Mazzocchi 2006). Discussions around the delegitimization of Indigenous knowledge and practices included lack of compensation for Indigenous knowledge holders, the displacement and loss of Indigenous knowledge (25), and the othering of Indigenous people (16). Authors discussed related epistemic concerns such as bioreduction (39, 41). Bioreduction in this context refers to the perception of a concept only in terms of its biochemical components, at the expense of subjective knowledge (Yaden et al. 2022). Articles suggested that bioreduction, which discounts subjective Indigenous knowledge and practices, may reduce the effectiveness of PAT and hinder understanding of its therapeutic properties (41) and that Indigenous traditional psychedelic users have “epistemic expertise” that should be respected (39).

Not all discussion of epistemic justice was specific to Indigenous people. Injustice was described where psychedelic-using communities’ views were discounted and seen as untrustworthy (41, 50). One article suggested that psychedelic-using communities, including for example artists, healers, and festival-goers, had “epistemic expertise” and thus should be consulted in research (39). Discussions around medicalization suggested that globalized Western psychiatric concepts displace diverse expressions of distress (24) and ignore systematic and social issues (37). Another epistemological concern was that PAT may cause “epistemological harm” because it had the potential to alter participants’ perception, cognition, and beliefs (23).

Suggestions for epistemic justice largely involved the adoption and acceptance of Indigenous psychedelic knowledge and practices. Articles suggested giving equal privilege to Indigenous and Western knowledge (24), learning from Indigenous knowledge holders, and privileging Indigenous ethics, practitioner responsibility, training procedures, and land repatriation (15, 24). One author suggested that the limitations of Western scientific knowledge should be acknowledged and that Indigenous approaches to epistemology could combine with Western knowledge to enhance research (15). Another author suggested that a system of mental health care should be developed that considers different epistemologies (7). Articles noted that spirituality was a knowledge system often closely linked to Indigenous psychedelic practice (24, 31). Several articles suggested that spirituality should be accepted as an aspect of PAT and that practitioners should have spiritual experiences as part of training (24, 31, 49). Regarding psychedelic-using communities, one article suggested the need to legitimize lived experience knowledge (39).

### Consultation

Discussion in the consultation workshop expanded upon previously identified ethical issues around informed consent, integrity, and reporting standards, access, and competence. Participants agreed that the ethical issues identified in the literature were consistent with their own experiences. They further highlighted the importance of several issues that they found pertinent. These included issues with informed consent during altered states, lack of open science in psychedelic research, and belief changes during PAT. Workshop participants also raised several issues not frequently discussed within the literature. These were 1) lack of post-trial care, 2) lack of consensus on models of care and competencies, and 3) how current research and its associated ethical issues will be translated into practice (see Table 3).

**Table 3:** Additional Themes Identified through Consultation

**Themes**	**Examples**
Lack of post-trial care	• Increased risk of suicidal behaviour for non-responders’ post-trial, compared to pre-trial. • How to support people who do not benefit from trials. • Those who go back to traditional therapy after psychedelic trials may be stigmatized or dismissed.
Lack of consensus on models of care and psychedelic practitioner competencies	• Therapist training is not regulated. • Widely used practices such as heteronormative dyads may not be suitable for all patient groups. • Whether practitioner experience/usage of psychedelics should be a requirement for competency.
How current research and its associated ethical issues will be translated into practice	• Conflicts of interest with prescribing. • Lack of discussion around rollout strategies. • Quality of care may decrease as access increases (e.g., ketamine clinics in the United States operating without sufficient ethical oversight).

## Discussion

### Standards of Practice and Integrity are Key Concerns

Overall, our findings reveal a broad coverage of ethical issues in psychedelic research and practice within the literature. The most frequently discussed issues related to standards of practice, including non-maleficence, competence, informed consent, and integrity. The focus on practical ethical issues is unsurprising, as it reflects core ethics concerns that are necessary to consider when undertaking research and clinical practice (Australian Psychological Society 2007). The overwhelming majority of articles included in our scoping review (n = 46; 90.2 %) came from developed, high-income countries (HICs). Further research would need to establish whether similar priorities were held by researchers from low- to middle-income countries.

### Characteristics of Competent Practice Remains Uncertain

Our findings revealed that, although competent practice is frequently discussed, there is no consensus on what constitutes competent practice in PAT, or how this will be established. Authors frequently described the importance of only practising within one’s scope of competence. Due to the increased vulnerability of participants during sessions, therapists without experience in the psychological and physiological effects of the psychedelic drug and without training in safely administering therapeutic touch may cause harm to participants (Rochester et al. 2022). Concern around the lack of any regulating body or credentialing system (Brennan et al. 2021) was expressed by workshop participants, who felt that this would lead to clinicians practising PAT without essential experience or training. This highlights a key issue for practice going forward: clear competency requirements are needed to ensure that patients receive a high standard of care.

One workshop participant with clinical trial expertise highlighted key issues regarding competent practice. Several aspects of PAT that are customary in practice are yet to be tested for their safety across varying patient groups (Okano et al. 2022; Wagner et al. 2019). First, although male-female therapist dyads are common practice for many psychedelic trials (Johnson et al. 2008), some workshop participants suggested that such dyads may cause harm or impede treatment for certain individuals, such as those with LGBTQIA+ identities, or non-traditional parenting structures (Wagner et al. 2019). Second, what constitutes a safe and comfortable setting for psychedelic sessions is generally agreed upon by psychedelic researchers (Okano et al. 2022). However, there is a lack of clarity as to how this might differ across participants (Okano et al. 2022). Research suggests that there is greater risk of adverse events if participants do not feel safe and supported in the research environment (Johnson et al. 2008). These findings elucidate two important areas of competence for further research: therapist dyads and psychedelic session setting.

Informed consent was discussed in depth within the literature. Authors identified the need for enhanced consent and provided practical recommendations. Workshop participants expanded on this discussion, highlighting several contingencies that consent protocols should account for. Participants expressed that it was important to explain to participants prior to the session that their ability to consent during the psychedelic session may be impaired and to outline contingencies for scenarios particularly involving therapist touch. However, conversely, there was a need to respect patient autonomy if they changed their mind during the session. These findings highlight the complexity of informed consent within PAT.

### Consultation and Collaboration with Indigenous People and Priority Populations

Several authors suggested that Indigenous methods or spiritual practices should be incorporated within PAT. However, despite discussion within the literature on the problematic appropriation of psychedelic knowledge, only two out of fifty-one articles consulted with Indigenous people or explicitly declared any Indigenous background. As noted by Celidwen et al (2023), Indigenous knowledge holders and practitioners should be actively included as leaders in deliberations related to the development of modern medical psychedelic research and practice. As such, future research should aim to consult knowledge holders on the incorporation of spiritual or shamanic aspects of PAT (Celidwen et al. 2023; Rochester et al. 2022).

There was also a significant lack of consultation with priority populations in the literature. This is despite widespread recognition of the critical role of collaboration and co-design in the development of an inclusive therapy that is accessible and effective for all communities (Close et al. 2021). None of the articles we analysed that discussed ethical issues concerning marginalized groups stated that they had consulted or collaborated with LGBTQIA+ people, people of colour, or those with disabilities, despite concerns that several of these groups may face significant barriers in accessing PAT (Michaels et al. 2018; Mintz et al. 2022). Relatedly, within our sample only nine articles employed empirical methods (such as literature reviews, collaboration, or interviews). This empirical gap has also been observed in ethical considerations of neurotechnology interventions, with authors expressing the need for more public engagement in order to develop robust ethical recommendations informed by real-world concerns (Jebari and Hansson 2013).

The recently published “ARC: A framework for access reciprocity and conduct” (Spriggs et al. 2023) provides a key example of how such collaboration may be achieved to inform ethical guidelines in PAT. Spriggs et al. (2023) aim to co-develop an ethics statement for access, reciprocity, and conduct with stakeholders from research, industry, therapy, lived experience, and Indigenous communities. Our study builds on this approach, presenting an example of how stakeholder engagement can be incorporated with findings from a comprehensive review to provide new insights in ethical issues in psychedelic research and practice.

### Who will Benefit from PAT, and Will These Benefits Continue Post-Trial?

Our findings reveal significant concerns around barriers to access for participants. Psychedelic trials employ strict exclusion criteria, with individuals excluded based on personal or family history of psychosis or the suspected or known presence of a pre-existing psychiatric condition, among other criteria (Appelbaum 2022). Authors within our sample recognized that such exclusion criteria were necessary to safeguard individuals against possible adverse effects of PAT, but also that this protection would exclude many individuals from benefitting. Further, those who are often excluded may be persons most likely to benefit from PAT, such as those with serious mental illness or suicidal ideation (Peterson and Sisti 2022). Authors did not provide recommendations in this area. Participants in our workshop conversely expressed the need to understand which patient groups are most susceptible to the risks of PAT in order to protect these individuals from potential risks. Given that participation in trials is necessary to build the evidence base for treating such individuals once access expands, further research is needed to understand and weigh the potential risks and benefits of PAT for these populations.

Although articles identified the need for research to directly benefit Indigenous populations, what these benefits would entail was rarely discussed. Benefit-sharing refers to the action of giving a portion of benefits derived from traditional knowledge to those knowledge holders (Hoffmann 2014). Celidwen et al (2023, 6) suggest that benefit-sharing should promote self-determination and “enable restitution of appropriate cultural, intellectual, religious, and spiritual property with the [free, prior informed consent] FPIC of Indigenous Nations.” This may involve for-profit psychedelic organizations sharing a percentage of royalties with traditional knowledge-holders, as well as funding community development projects (Wynberg 2004). Outside of those highlighted by Celidwen et al. (2023), guidelines or practices for ensuring that Indigenous populations benefit from psychedelic research and receive appropriate reparations were not discussed in the literature. Such practice guidelines are urgently needed.

Our findings revealed that post-trial care was a neglected issue in the literature. Although the importance of equitable access to PAT was frequently discussed, only one article questioned whether participants would continue to receive benefits from treatment after the conclusion of the trial. Post-trial care aims to provide participants who have experienced benefits during a trial with continued benefits from that treatment after the trial has ended, in order to avoid exploitation of people who may not have access to appropriate community care, and/or are at risk of relapse (Sofaer and Strech 2011). The urgent need for clear ethical standards for the provision of post-trial care was highlighted during our consultation. Many participants of psychedelic trials have intractable or treatment-resistant illnesses (Smith and Appelbaum 2022). As one workshop participant noted, those who do not respond to treatment are left without options and may be at higher risk of suicide after a trial. Indeed, cases of suicide among participants who fail to respond have been reported in trials of other experimental interventions, such as deep brain stimulation (Gilbert 2013). Further, as noted by workshop participants, those who go back to more traditional healthcare treatments after a PAT trial may be stigmatized or discriminated against by therapists who are sceptical about PAT. In addition, their therapist may not be adequately trained to be able to provide therapy to someone who has undergone PAT. The need for post-trial care and what exactly this should entail is debated (Cook et al. 2016; Jacobs et al. 2023). However, given barriers to accessing PAT, as well as the harm participants may face during or after the trial, the lack of discussion noted within this review highlights a significant need for further research.

### Challenges to the Ethical Translation of Psychedelic Innovation

Access to PAT is increasing rapidly (Smith and Appelbaum 2021), and as such it is crucial to consider how clinical trial research will translate into practice. Several key ethical translational concerns were noted by workshop participants that were neglected within the literature. As access to psychedelic-assisted therapies and for-profit clinics increase, quality of care for patients is likely to decrease. In the United States, off-label ketamine use is unregulated and for-profit ketamine clinics have proliferated to meet public demand (Smith and Appelbaum 2021). Many ketamine clinics make unsubstantiated claims regarding the evidence for ketamine use and treat indications for which there is a lack of robust clinical evidence (Smith and Appelbaum 2021). Often patients are not adequately screened for medical need and do not receive any follow-up care (Smith and Appelbaum 2021). Those with conflicts of interest in prescribing may be less likely to adhere to standards of care (Manoharan et al. 2022). As one participant noted, problematic medicinal cannabis prescribing practices such as providing prescriptions to those who are at risk of adverse reactions may extend to PAT (MacPhail et al. 2022).

## Limitations

Our study identified key ethical issues discussed within the literature. However, how frequently issues were mentioned or discussed is not necessarily a reflection of their ethical or moral significance. The consultation workshop aimed to address this limitation by exploring their relevance to persons with expertise and experience in psychedelic research or practice. Participants also identified several key issues that were neglected within the literature. Future research could further prioritize these ethical issues by conducting consensus workshops, Delphi studies or participatory design methods. These approaches can provide direction on research and usually recruit practitioners as well as researchers in order to best understand how research translates into practice (Verbeten 2021).

Although our study sought to capture whose views were being represented within the data, diversities within authorship may have been present that were not identified. Analysis of authorship relied on declarations of institutional affiliation within each article, and as such any information that was not explicitly declared will not have been included. Geographic location was determined based on the affiliations of the first author. While first authors typically lead decisions surrounding the article, and as such are generally representative of viewpoints within the article (Bhattacharya 2010), there may have been diversities within authorship that were not identified.

Further, our study did not include grey literature. As such relevant viewpoints from organizational and government agencies may not be represented in our findings. This limitation was mitigated in part through citation chaining and snowball sampling.

## Conclusion

Our scoping review provides several insights into the ethical issues raised by psychedelic research and practice. Findings reveal a broad coverage of ethical issues and recommendations discussed within the literature. However, there were several key issues that were overlooked, highlighting the need for further research. Our consultation workshop provided insights into areas of practice where discussion was limited, such as the lack of any clear competencies for therapists and the potential harms associated with heteronormative therapist dyads. Despite the importance of collaboration in establishing ethical guidelines that are safe and effective for all (Close et al. 2021), we found a significant lack of collaboration with those with lived experience and Indigenous and other cultural minorities in the literature. Our findings also reveal a lack of consideration for post-trial care in psychedelic research, despite the considerable risks associated with PAT for certain individuals. Lastly, several translational issues must be addressed to ensure that conflicts of interest and hype do not take precedence over quality of care. The considerations outlined by this review may inform future ethical guidelines for psychedelic research and practice and are essential to prioritizing patient wellbeing and safety as access to PAT expands.

## Supplementary Information

Below is the link to the electronic supplementary material.

Supplementary file1 (DOCX 821 KB)

## Data Availability

Collected articles can be accessed under Supplementary Material D.
