# Supplementary material for: Ethical Issues and Recommendations in Psychedelic Research and Practice: A Scoping Review
Source: J Bioeth Inq. 2025 Aug 7;23(1):49–65. doi: 10.1007/s11673-025-10454-3 (PMC13068691; doi:10.1007/s11673-025-10454-3)
Supplement: Supplementary file 1 — Supplementary file1 (DOCX 821 KB) [file 11673_2025_10454_MOESM1_ESM.docx]

**Supplementary Materials**

**Journal of Bioethical Inquiry**

**Ethical Issues and Recommendations in Psychedelic Research and Practice: A Scoping Review**

**Brittain, N.,^1^ Higgins, N.,^1,3^ Barber, M.,^1^ Choi, W.,^2^ Carter, A.,^1,3*^ and Gardner, J.^3*^**

**Brittain. N**

School of Psychological Sciences, Monash University

770 Blackburn Rd, Clayton, VIC

Melbourne, 3800 AUSTRALIA

Email: [noa.brittain@gmail.com](mailto:noa.brittain@gmail.com)

**Higgins. N**

School of Psychological Sciences, Monash University

770 Blackburn Rd, Clayton, VIC

Melbourne, 3800 AUSTRALIA

Email: [nathan.higgins@monash.edu](mailto:nathan.higgins@monash.edu)

**Barber. M**

School of Psychological Sciences, Monash University

770 Blackburn Rd, Clayton, VIC

Melbourne, 3800 AUSTRALIA

Email: [Michaela.Barber@monash.edu](mailto:Michaela.Barber@monash.edu)

**Choi. W**

Brown University

Providence, RI 02912

UNITED STATES

Email: [william_choi@brown.edu](mailto:william_choi@brown.edu)

**Carter. A**

Monash Bioethics Centre and the School of Philosophical, Historical and International Studies

School of Psychological Sciences, Monash University

770 Blackburn Rd, Clayton, VIC

Melbourne, 3800 AUSTRALIA

Email: [adrian.carter@monash.edu](mailto:adrian.carter@monash.edu)

**Gardner. J**

Monash Bioethics Centre and the School of Philosophical, Historical and International Studies

20 Chancellors Walk, Clayton, VIC

Melbourne, 3800 AUSTRALIA

Email: [john.gardner@monash.edu](mailto:john.gardner@monash.edu)

# Supplementary Material A

**
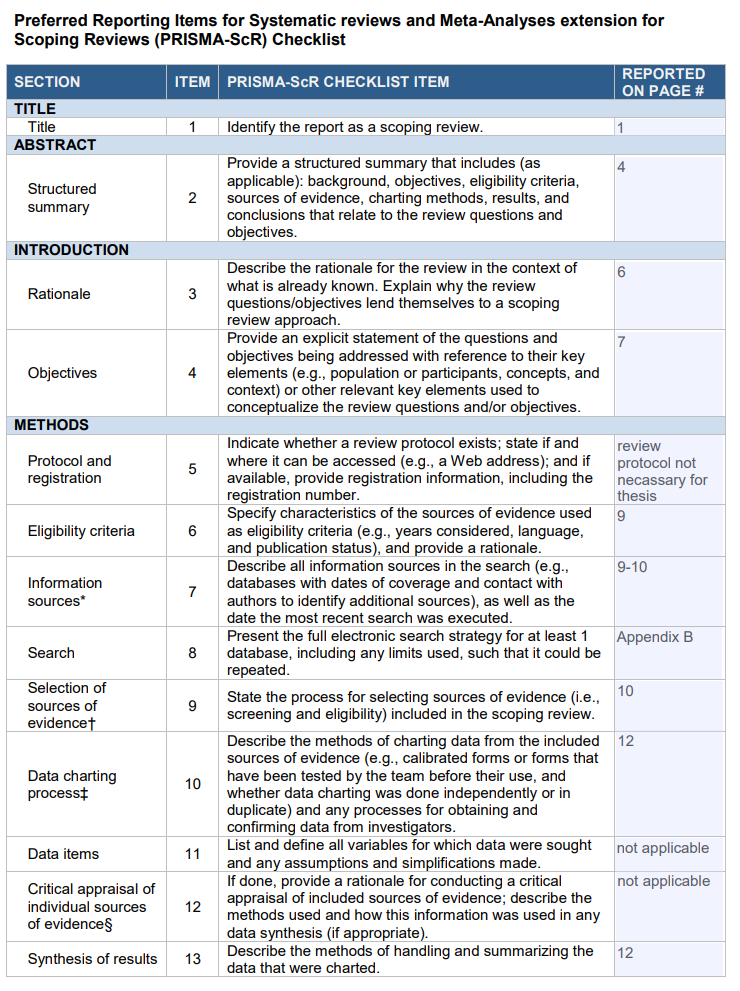
**

**
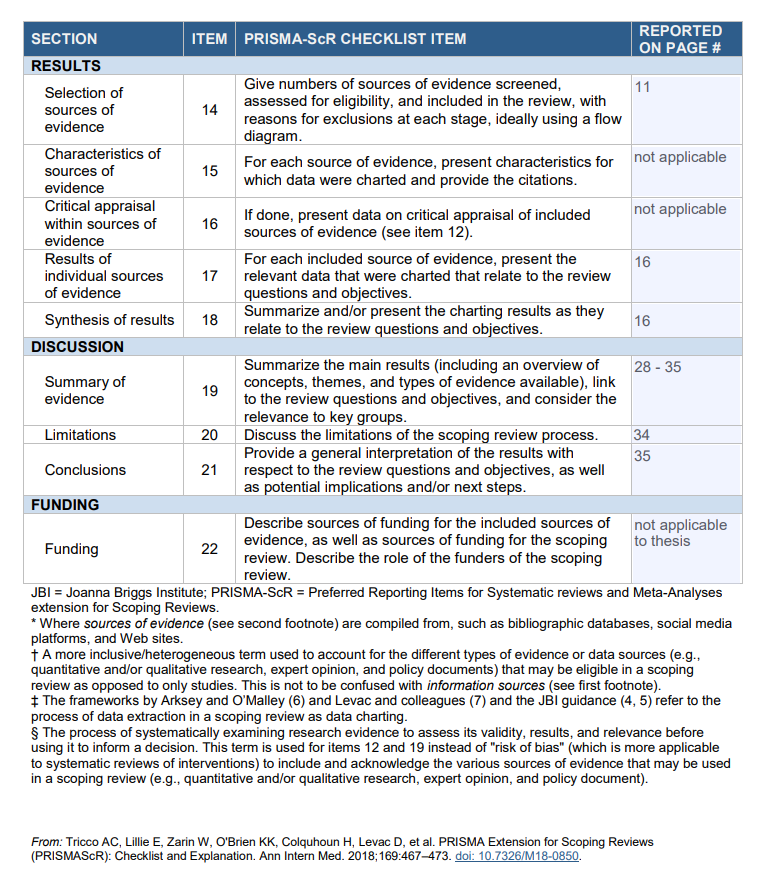
**

# Supplementary Material B

APA PsycInfo

1 psychedelic drugs/ or bufotenine/ or ketamine/ or lysergic acid diethylamide/ or mescaline/ or methylenedioxymethamphetamine/ or psilocybin/ 8343

2 exp Ethics/ 61160

3 ethic*.mp. [mp=title, abstract, heading word, table of contents, key concepts, original title, tests & measures, mesh word] 100966

4 Treatment Guidelines/ 9463

5 framework*.mp. [mp=title, abstract, heading word, table of contents, key concepts, original title, tests & measures, mesh word] 243846

6 guideline*.mp. [mp=title, abstract, heading word, table of contents, key concepts, original title, tests & measures, mesh word] 85153

7 (competenc* or transparency).mp. [mp=title, abstract, heading word, table of contents, key concepts, original title, tests & measures, mesh word] 128023

8 psychedeli*.mp. [mp=title, abstract, heading word, table of contents, key concepts, original title, tests & measures, mesh word] 3058

9 hallucinogen*.mp. [mp=title, abstract, heading word, table of contents, key concepts, original title, tests & measures, mesh word] 4420

10 Psychedelic Assisted Therapy/ 75

11 ayahuasca/ or N,N-Dimethyltryptamine/ or ibogaine.mp. [mp=title, abstract, heading word, table of contents, key concepts, original title, tests & measures, mesh word] 193

12 1 or 8 or 9 or 10 or 11 11209

13 2 or 3 or 4 or 5 or 6 or 7 521223

14 12 and 13 464

#
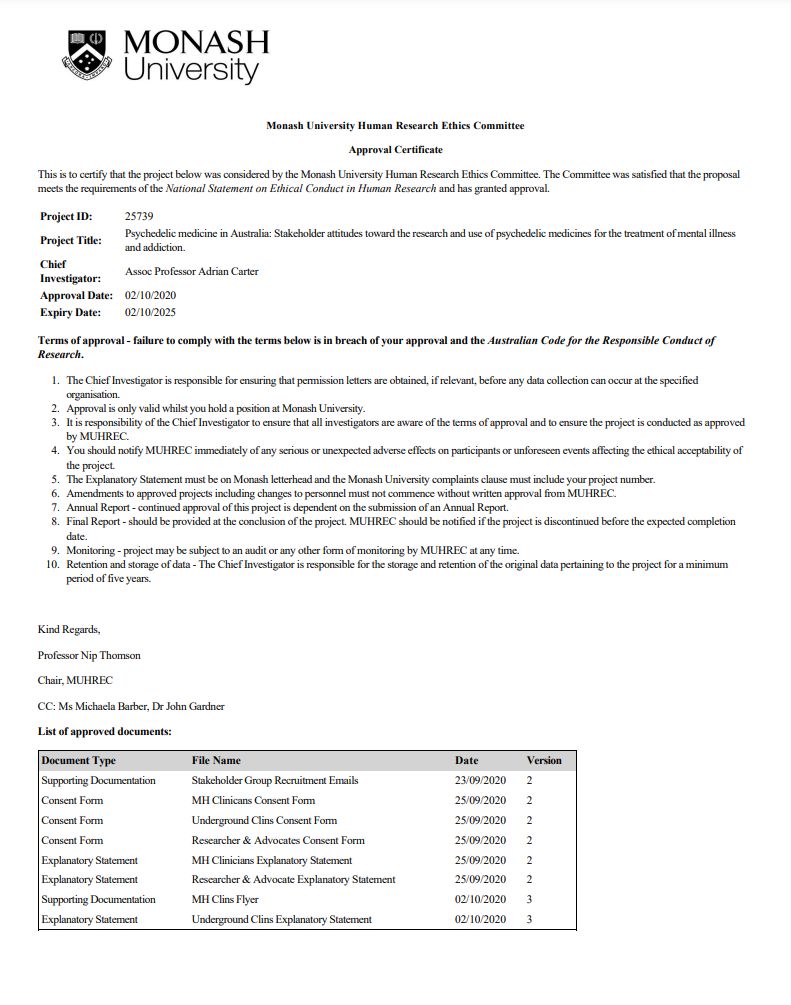
 Supplementary Material C

# Supplementary Material D

1. Aday, J. S., Carhart-Harris, R. L., & Woolley, J. D. (2023). Emerging Challenges for Psychedelic Therapy. *JAMA Psychiatry*. <https://doi.org/10.1001/jamapsychiatry.2023.0549>
2. Anderson, B. T., Danforth, A. L., & Grob, C. S. (2020). Psychedelic medicine: Safety and ethical concerns [Clinical Psychopharmacology 3340]. *The Lancet Psychiatry*, *7*(10), 829-830. https://doi.org/https://dx.doi.org/10.1016/S2215-0366%2820%2930146-2
3. Appelbaum, P. S. (2022). Psychedelic research and the real world [Article]. *Nature*, *609*(7929), S95. https://doi.org/10.1038/d41586-022-02875-6
4. Belouin, S. J., Averill, L. A., Henningfield, J. E., Xenakis, S. N., Donato, I., Grob, C. S., Berger, A., Magar, V., Danforth, A. L., & Anderson, B. T. (2022). Policy considerations that support equitable access to responsible, accountable, safe, and ethical uses of psychedelic medicines [Psychopharmacology 2580]. *Neuropharmacology*, *219*, 1-6. https://doi.org/https://dx.doi.org/10.1016/j.neuropharm.2022.109214 (International Journal of Neuropharmacology)
5. Beswerchij, A., & Sisti, D. (2022). From Underground to Mainstream: Establishing a Medical Lexicon for Psychedelic Therapy [Article]. *Frontiers in Psychiatry*, *13*, Article 870507. https://doi.org/10.3389/fpsyt.2022.870507
6. Bodnár, K. J., & Kakuk, P. (2019). Research ethics aspects of experimentation with LSD on human subjects: a historical and ethical review [Review]. *Medicine, Health Care and Philosophy*, *22*(2), 327-337. https://doi.org/10.1007/s11019-018-9871-9
7. Bouso, J. C., & Sánchez-Avilés, C. (2020). Traditional healing practices involving psychoactive plants and the global mental health agenda: Opportunities, pitfalls, and challenges in the “right to science” framework [Article]. *Health and Human Rights*, *22*(1), 145-150. https://www.scopus.com/inward/record.uri?eid=2-s2.0-85086944346&partnerID=40&md5=170692c243954ebcf428e99ce190d53f
8. Brennan, W., & Belser, A. B. (2022). Models of Psychedelic-Assisted Psychotherapy: A Contemporary Assessment and an Introduction to EMBARK, a Transdiagnostic, Trans-Drug Model [Article]. *Frontiers in Psychology*, *13*, Article 866018. https://doi.org/10.3389/fpsyg.2022.866018
9. Brennan, W., Jackson, M. A., MacLean, K., & Ponterotto, J. G. (2021). A Qualitative Exploration of Relational Ethical Challenges and Practices in Psychedelic Healing [Article]. *Journal of Humanistic Psychology*. https://doi.org/10.1177/00221678211045265
10. Buchanan, N. T. (2021). Ensuring the psychedelic renaissance and radical healing reach the Black community: Commentary on Culture and Psychedelic Psychotherapy. *Journal of Psychedelic Studies*, *4*(3), 142-145. https://doi.org/https://doi.org/10.1556/2054.2020.00145
11. Calder, A., & Hasler, G. (2023). Extrapharmacological Safety Topics in Psychedelic-Assisted Psychotherapy. *JAMA Psychiatry*. https://doi.org/10.1001/jamapsychiatry.2023.1031
12. Campbell, M., & Williams, M. T. (2021). The Ethic of Access: An AIDS Activist Won Public Access to Experimental Therapies, and This Must Now Extend to Psychedelics for Mental Illness [Article]. *Frontiers in Psychiatry*, *12*, Article 680626. https://doi.org/10.3389/fpsyt.2021.680626
13. Celidwen, Y., Redvers, N., Githaiga, C., Calambás, J., Añaños, K., Chindoy, M. E., Vitale, R., Rojas, J. N., Mondragón, D., Rosalío, Y. V., & Sacbajá, A. (2023). Ethical principles of traditional Indigenous medicine to guide western psychedelic research and practice [Review]. *Lancet Regional Health - Americas*, *18*, Article 100410. https://doi.org/10.1016/j.lana.2022.100410
14. Close, J. B., Bornemann, J., Piggin, M., Jayacodi, S., Luan, L. X., Carhart-Harris, R., & Spriggs, M. J. (2021). Co-design of Guidance for Patient and Public Involvement in Psychedelic Research [Article]. *Frontiers in Psychiatry*, *12*, Article 727496. https://doi.org/10.3389/fpsyt.2021.727496
15. Fotiou, E. (2020). The role of Indigenous knowledges in psychedelic science [Clinical Psychopharmacology 3340]. *Journal of Psychedelic Studies*, *4*(1), 16-23. https://doi.org/https://dx.doi.org/10.1556/2054.2019.031
16. George, J. R., Michaels, T. I., Sevelius, J., & Williams, M. T. (2020). The psychedelic renaissance and the limitations of a White-dominant medical framework: A call for indigenous and ethnic minority inclusion [Clinical Psychopharmacology 3340]. *Journal of Psychedelic Studies*, *4*(1), 4-15. https://doi.org/https://dx.doi.org/10.1556/2054.2019.015
17. Gerber, K., Flores, I. G., Ruiz, A. C., Ali, I., Ginsberg, N. L., & Schenberg, E. E. (2021). Ethical Concerns about Psilocybin ual Property. *ACS Pharmacology & Translational Science*, *4*(2), 573-577. https://doi.org/10.1021/acsptsci.0c00171
18. Hall, W. D., & Humphreys, K. (2022). Is good science leading the way in the therapeutic use of psychedelic drugs? *Psychological Medicine*, *52*(14), 2849-2851. https://doi.org/10.1017/S0033291722003191
19. Hauskeller, C., Artinian, T., Fiske, A., Schwartz Marin, E., González Romero, O. S., Luna, L. E., Crickmore, J., & Sjöstedt-Hughes, P. (2022). Decolonization is a metaphor towards a different ethic. The case from psychedelic studies [Article]. *Interdisciplinary Science Reviews*. https://doi.org/10.1080/03080188.2022.2122788
20. Jacobs, E. (2023). Transformative experience and informed consent to psychedelic-assisted psychotherapy [Hypothesis and Theory]. *Frontiers in Psychology*, *14*. https://doi.org/10.3389/fpsyg.2023.1108333
21. Johnson, M. W. (2021). Consciousness, Religion, and Gurus: Pitfalls of Psychedelic Medicine [Review]. *ACS Pharmacology and Translational Science*, *4*(2), 578-581. https://doi.org/10.1021/acsptsci.0c00198
22. Kious, B., Schwartz, Z., & Lewis, B. (2023). Should we be leery of being Leary? Concerns about psychedelic use by psychedelic researchers [Psychopharmacology 2580]. *Journal of Psychopharmacology*, *37*(1), 45-48. https://doi.org/https://dx.doi.org/10.1177/02698811221133461
23. Letheby, C., & Mattu, J. (2021). Philosophy and classic psychedelics: A review of some emerging themes [Psychopharmacology 2580]. *Journal of Psychedelic Studies*, *5*(3), 166-175. https://doi.org/https://dx.doi.org/10.1556/2054.2021.00191
24. Marcus, O. (2022). ‘Everybody’s creating it along the way’: ethical tensions among globalized ayahuasca shamanisms and therapeutic integration practices [Article]. *Interdisciplinary Science Reviews*. https://doi.org/10.1080/03080188.2022.2075201
25. McMillan, R. M. (2021). Global bioethical challenges of medicalising psychedelics [Clinical Psychopharmacology 3340]. *Journal of Psychedelic Studies*, *5*(2), 57-64. http://ovidsp.ovid.com/ovidweb.cgi?T=JS&PAGE=reference&D=psyc20&NEWS=N&AN=2022-06987-001
26. Michaels, T. I., Purdon, J., Collins, A., & Williams, M. T. (2018). Inclusion of people of color in psychedelic-assisted psychotherapy: a review of the literature. *BMC Psychiatry*, *18*(1). https://doi.org/ 10.1186/s12888-018-1824-6
27. Mintz, K. T., Gammer, B., Khan, A. J., Shaub, G., Levine, S., & Sisti, D. (2022). Physical Disability and Psychedelic Therapies: An Agenda for Inclusive Research and Practice [Article]. *Frontiers in Psychiatry*, *13*, Article 914458. https://doi.org/10.3389/fpsyt.2022.914458
28. Morales, J., Quan, E., Arshed, A., & Jordan, A. (2022). Racial Disparities in Access to Psychedelic Treatments and Inclusion in Research Trials [Article]. *Psychiatric Annals*, *52*(12), 494-499. https://doi.org/10.3928/00485713-20221123-01
29. Ona, G., Kohek, M., & Bouso, J. C. (2022). The illusion of knowledge in the emerging field of psychedelic research [Clinical Psychopharmacology 3340]. *New Ideas in Psychology*, *67*, 1-6. https://doi.org/https://dx.doi.org/10.1016/j.newideapsych.2022.100967
30. Ortiz, C. E., Dourron, H. M., Sweat, N. W., Garcia-Romeu, A., MacCarthy, S., Anderson, B. T., & Hendricks, P. S. (2022). Special considerations for evaluating psilocybin-facilitated psychotherapy in vulnerable populations [Psychotherapy & Psychotherapeutic Counseling 3310]. *Neuropharmacology*, *214*, 1-5. https://doi.org/https://dx.doi.org/10.1016/j.neuropharm.2022.109127 (International Journal of Neuropharmacology)
31. Penn, A. D., Phelps, J., Rosa, W. E., & Watson, J. (2021). Psychedelic-Assisted Psychotherapy Practices and Human Caring Science: Toward a Care-Informed Model of Treatment [Article]. *Journal of Humanistic Psychology*. https://doi.org/10.1177/00221678211011013
32. Peterson, A., Largent, E. A., Lynch, H. F., Karlawish, J., & Sisti, D. (2023). Journeying to Ixtlan: Ethics of Psychedelic Medicine and Research for Alzheimer's Disease and Related Dementias. *AJOB neuroscience*, *14*(2), 107-123. https://doi.org/https://dx.doi.org/10.1080/21507740.2022.2148771
33. Peterson, A., & Sisti, D. (2022). Skip the Trip? Five Arguments on the Use of Nonhallucinogenic Psychedelics in Psychiatry. *Cambridge Quarterly of Healthcare Ethics*, *31*(4), 472-476. https://www.proquest.com/docview/2737329006?accountid=12528&bdid=35168&_bd=VxN6%2FuyL2%2BXUaus%2BARmysIEz99U%3D
34. Peterson, A., Tagliazucchi, E., & Weijer, C. (2019). The ethics of psychedelic research in disorders of consciousness [Psychological Disorders 3210]. *Neuroscience of Consciousness*, *2019*(1). https://doi.org/https://dx.doi.org/10.1093/nc/niz013
35. Petranker, R., Anderson, T., & Farb, N. (2020). Psychedelic research and the need for transparency: Polishing Alice's looking glass [Clinical Psychopharmacology 3340]. *Frontiers in Psychology*, *11*. https://doi.org/https://dx.doi.org/10.3389/fpsyg.2020.01681
36. Phelps, J., & Henry, J. (2022). Foundations for Training Psychedelic Therapists. In *Current Topics in Behavioral Neurosciences* (Vol. 56, pp. 93-109): Springer Science and Business Media Deutschland GmbH.
37. Plesa, P., & Petranker, R. (2022). Manifest your desires: Psychedelics and the self-help industry [Article]. *International Journal of Drug Policy*, *105*, Article 103704. https://doi.org/10.1016/j.drugpo.2022.103704
38. Rasmussen, K., & Olson, D. E. (2022). Psychedelics as Standard of Care? Many Questions Remain. *Cambridge Quarterly of Healthcare Ethics*, *31*(4), 477-481.
39. Riccardo Miceli, M. (2022). Psychedelic injustice: should bioethics tune in to the voices of psychedelic-using communities? *Medical Humanities*, *48*(3), 269-272. https://www.proquest.com/docview/2705404525?accountid=12528&bdid=35168&_bd=ftccSrr54x%2FQN%2BYVJ7x2RT4Exm8%3D
40. Rochester, J., Vallely, A., Grof, P., Williams, M. T., Chang, H., & Caldwell, K. (2022). Entheogens and psychedelics in Canada: Proposal for a new paradigm [Substance Abuse & Addiction 3233]. *Canadian Psychology / Psychologie canadienne*, *63*(3), 413-430. https://doi.org/https://dx.doi.org/10.1037/cap0000285
41. Schenberg, E. E., & Gerber, K. (2022). Overcoming epistemic injustices in the biomedical study of ayahuasca: Towards ethical and sustainable regulation [Article]. *Transcultural Psychiatry*, *59*(5), 610-624. https://doi.org/10.1177/13634615211062962
42. Smith, D. T., Faber, S. C., Buchanan, N. T., Foster, D., & Green, L. (2022). The need for psychedelic-assisted therapy in the Black community and the burdens of its provision. *Frontiers in Psychiatry Vol 12 2022, ArtID 774736*, *12*. https://doi.org/https://dx.doi.org/10.3389/fpsyt.2021.774736
43. Smith, W. R., & Appelbaum, P. S. (2022). Novel ethical and policy issues in psychiatric uses of psychedelic substances [Substance Abuse & Addiction 3233]. *Neuropharmacology*, *216*, 1-5. https://doi.org/https://dx.doi.org/10.1016/j.neuropharm.2022.109165 (International Journal of Neuropharmacology)
44. Smith, W. R., & Sisti, D. (2021). Ethics and ego dissolution: The case of psilocybin [Professional Ethics & Standards & Liability 3450]. *Journal of Medical Ethics: Journal of the Institute of Medical Ethics*, *47*(12), 807-814. https://doi.org/https://dx.doi.org/10.1136/medethics-2020-106070
45. Spriggs, M. J., Murphy-Beiner, A., Murphy, R., Bornemann, J., Thurgur, H., & Schlag, A. K. (2023). ARC: a framework for access, reciprocity and conduct in psychedelic therapies [Original Research]. *Frontiers in Psychology*, *14*. https://doi.org/10.3389/fpsyg.2023.1119115
46. Strauss, D., De La Salle, S., Sloshower, J., & Williams, M. T. (2021). Research abuses against people of colour and other vulnerable groups in early psychedelic research [Article]. *Journal of Medical Ethics*, *42*(11), Article medethics-2021-107262. https://doi.org/10.1136/medethics-2021-107262
47. Villiger, D., & Trachsel, M. (2023). With great power comes great vulnerability: an ethical analysis of psychedelics’ therapeutic mechanisms proposed by the REBUS hypothesis. *Journal of Medical Ethics*, jme-2022-108816. https://www.proquest.com/docview/2799820569?accountid=12528&bdid=35168&_bd=Eo4HOqpPd8vtxcHuwJ5O349JLiI%3D
48. Wallace, B., & Rea, K. (2021). Enhancing equity-oriented care in psychedelic medicine: Utilizing the EQUIP framework [Article]. *International Journal of Drug Policy*, *98*, Article 103429. https://doi.org/10.1016/j.drugpo.2021.103429
49. Waring, D. R. (2023). Opening Death’s Door: Psilocybin and Existential Suffering in Palliative Care. In *Philosophy and Medicine* (Vol. 132, pp. 235-262): Springer Science and Business Media B.V.
50. Yaden, D. B., Earp, B. D., & Griffiths, R. R. (2022). Ethical Issues Regarding Nonsubjective Psychedelics as Standard of Care [Article]. *Cambridge Quarterly of Healthcare Ethics*, *31*(4), 464-471. https://doi.org/10.1017/S096318012200007X
51. Yaden, D. B., Potash, J. B., & Griffiths, R. R. (2022). Preparing for the Bursting of the Psychedelic Hype Bubble [Note]. *JAMA Psychiatry*, *79*(10), 943-944. https://doi.org/10.1001/jamapsychiatry.2022.2546
